# Text Messaging Versus Email Messaging to Support Patients With Major Depressive Disorder: Protocol for a Randomized Hybrid Type II Effectiveness-Implementation Trial

**DOI:** 10.2196/29495

**Published:** 2021-10-13

**Authors:** Medard Kofi Adu, Reham Shalaby, Ejemai Eboreime, Adegboyega Sapara, Nnamdi Nkire, Rajan Chawla, Chidi Chima, Michael Achor, Felix Osiogo, Pierre Chue, Andrew J Greenshaw, Vincent Israel Agyapong

**Affiliations:** 1 Department of Psychiatry Faculty of Medicine and Dentistry University of Alberta Edmonton, AB Canada; 2 Department of Psychiatry Faculty of Medicine Dalhousie University Halifax, NS Canada

**Keywords:** email messaging, text messaging, supportive, major depressive disorder, randomized trial, mental health, digital health, mobile health, mHealth, patient care, health policy, decision-making, health care resources

## Abstract

**Background:**

Major depressive disorder (MDD) accounts for 40.5% of disability-adjusted life years caused by mental and substance use disorders. Barriers such as stigma and financial and physical access to care have been reported, highlighting the need for innovative, accessible, and cost-effective psychological interventions. The effectiveness of supportive SMS text messaging in alleviating depression symptoms has been proven in clinical trials, but this approach can only help those with mobile phones.

**Objective:**

This paper presents the protocol for a study that will aim to evaluate the feasibility, comparative effectiveness, and user satisfaction of daily supportive email messaging as an effective strategy compared to daily supportive text messaging as part of the treatment of patients with MDD.

**Methods:**

This trial will be carried out using a hybrid type II implementation-effectiveness design. This design evaluates the effectiveness of an implementation strategy or intervention, while also evaluating the implementation context associated with the intervention. Patients with MDD receiving usual care will be randomized to receive either daily supportive email messaging or daily supportive text messaging of the same content for 6 months. The Patient Health Questionnaire-9, the Generalized Anxiety Disorder-7, and the 5-item World Health Organization Well-Being Index will be used to evaluate the effectiveness of both strategies. The implementation evaluation will be guided by the RE-AIM (Reach, Effectiveness, Adoption, Implementation, and Maintenance) framework, as well as the Consolidated Framework for Implementation Research. All outcome measures will be analyzed using descriptive and inferential statistics. Qualitative data will be analyzed using thematic analysis.

**Results:**

Data collection for this trial began in April 2021. We expect the study results to be available within 18 months of study commencement. The results will shed light on the feasibility, acceptability, and effectiveness of using automated emails as a strategy for delivering supportive messages to patients with MDD in comparison to text messaging.

**Conclusions:**

The outcome of this trial will have translational impact on routine patient care and access to mental health, as well as potentially support mental health policy decision-making for health care resource allocation.

**Trial Registration:**

ClinicalTrials.gov NCT04638231; https://clinicaltrials.gov/ct2/show/NCT04638231

**International Registered Report Identifier (IRRID):**

DERR1-10.2196/29495

## Introduction

### Background

Depression is a debilitating condition characterized by changes in mood, self-attitude, cognitive functioning, sleep, appetite, and energy levels [1,2]. Decreases in the quality of life associated with depression leads to impairment in occupational and social functioning [2,3]. In 2010, it was estimated that mental and substance use disorders were the leading cause of years lived with disability worldwide, with depressive disorders accounting for 40.5% of disability-adjusted life years caused by mental and substance use disorders [4]. Depression is thus a major contributor to the overall global burden of disease [4-6], and it is projected by the World Health Organization that major depressive disorder (MDD) will be the leading cause of disability worldwide by 2030 [7].

In 2012, over 3.2 million Canadians over 15 years of age (11.3%) reported symptoms of major depression, which is associated with higher health and service utilization than most other patients [8]. Psychosocial interventions and pharmacotherapy are among preferred first-line treatments for severe mental health problems including major depression [9]. Psychotherapies, such as cognitive behavior therapy, interpersonal psychotherapy, problem-solving, and behavioral activation are common and effective forms of treatment for depression [9]. However, access to these psychotherapies is limited by human resource capacity constraints, which leaves the majority of people with depressive disorders untreated [10,11]. Access is further limited by geographic location as psychosocial services are also more frequently available in cities and towns [12], with far less access for rural inhabitants [13]. Even in cities and towns, these services are mostly only available during the working days and day-time business hours [8,14], with caregivers often dealing with twice the recommended number of clients, further restricting appointment availability [14]. Long wait-times to access counseling services and the stigma associated with seeking mental health counseling also compound the problem. In the 2012 Canadian Community Health Survey on Mental Health, barriers such as lack of a readily available care system, stigma, and affordability of health care services were reported by 2.3 million Canadians, who expressed they had unmet or partially met mental health care needs [15,16]. It is clear that the traditional ways of providing mental health care alone will not be able to meet the demands for services given that the prevalence of depression is so high and not likely to decrease any time soon [17]. Consequently, there is a need to develop innovative psychological interventions that are not human resource intensive, and are easily accessible, cost-effective, independent of geographic location, scalable, and can be offered to thousands of people simultaneously.

Digital technologies for the provision of health care interventions have advanced significantly in the last decade, and further development of this field looks very promising [18]. Current evidence supports the efficacy and cost-effectiveness of these new technologies, such as tele–mental health, as they may enhance access to mental health care and contribute to closing the treatment gap that has existed over the years [19,20]. Useful communication methods for the delivery of mental health services have included smartphone apps, text messages, and email [20,21]. There is therefore a need for the health profession to embrace these new trends of digital technological care, particularly in the domain of mental health [22].

### Intervention and Implementation Strategies

eHealth approaches have been rapidly expanding in recent decades, with evidence indicating that the provision of internet-based mental health services is clinically effective [23] and cost-effective even though many health professionals have, until quite recently, been slow to engage in this clinical domain [24,25]. Supportive text messages have also become increasingly accepted as an appropriate and acceptable means of delivering psychological care to patients with mental health issues [26]. It is estimated that 99% of received text messages are opened, and 90% of all text messages are read within 3 minutes of reception [27], presenting an accessible opportunity to close the psychological treatment gap for patients with depression [28]. In 3 randomized controlled trials conducted in Ireland [29] and Canada [30], patients with MDD who received twice-daily supportive text messages had significantly greater reductions in their depression symptom scores than patients who received treatment as usual. In the first of the 2 studies conducted in Ireland, after 3 months, the mean difference in Becks Depression Inventory-II (BDI-II) scores between the intervention and control groups was –7.9 (95% CI –13.06 to –2.76, Cohen *d*=0.85) in favor of the intervention group [29]. Similar results were reported in another Irish study with a larger sample size and, after 3 months of exposure, a Canadian study reported a significant difference in mean BDI-II scores for the intervention versus control groups (mean 20.8, SD 11.7 vs mean 24.9, SD 11.5, respectively; *F*_1,60_=4.83, *P*=.03, *η*_p_^2^=0.07) with an effect size (Cohen *d*) of 0.67 [30]. A recent literature review of studies conducted on the effectiveness of text messaging as an adjunct therapy for mothers with postpartum depression living in low-income countries also reported a positive outcome [31], where the mothers showed a preference for receiving psychological care via text messaging [32]. Several studies reported high user satisfaction with a supportive text message intervention [28,33], and in one of these studies, 83% of subscribers to the Text4Mood program reported that daily messages contributed to improving their overall mental well-being [28].

Though patronage of supportive text messaging programs is deemed great in most cases due to the high numbers of subscribers, anecdotally, some people were unable to subscribe to supportive text messaging programs such as Text4Hope [33] and Text4Mood [28] because they did not have active mobile phone numbers, including several individuals for whom these programs were recommended by Addiction and Mental Health clinics in Edmonton resulting in inquiries as to whether the messages could be sent to them via email. In the participants’ satisfaction survey for the Text4Hope program, the majority of respondents (64%) were in favor of email messaging as part of their health care support during crisis periods despite having access to mobile phones.

With the rise of eHealth apps, email has been established as a secure avenue of communication, increasingly used for interactions between physicians and their patients [34]. The uniqueness of email messaging can be linked to its special characteristics, including flexibility of message length, asynchronous communication, and rapid message delivery [18]. There is a lack of data on whether email messaging is as effective as text messaging to clinically support patients with MDD.

### Study Aims and Objectives

The *Supportive Text vs Email Messaging* (STEM) trial aims to evaluate comparatively the implementation and impact of two implementation strategies (text messaging and email messaging) for delivering supportive messages to patients with MDD.

The specific objectives are outlined below:

1. Implementation evaluation:
  - To compare the impact of both strategies for delivering the daily supportive messages on implementation outcomes (adoption, fidelity, reach, cost, sustainability);
  - To evaluate contextual factors that could inhibit or facilitate the implementation of each strategy.
2. Clinical effectiveness evaluation:
  - To compare the mean difference in Patient Health Questionnaire-9 (PHQ-9) [35] scores from baseline and at 6, 12, and 24 weeks for patients with MDD receiving standard care plus daily supportive email messages to those receiving standard care plus daily supportive text messages;
  - To compare the quality of life using the 5-item World Health Organization Well-Being Index (WHO-5) [36] at baseline and at 6, 12, and 24 weeks for patients with MDD receiving standard care plus daily supportive email messages to those receiving standard care plus daily supportive text messages;
  - To compare anxiety levels using the Generalized Anxiety Disorder-7 (GAD-7) scale [37], at baseline and at 6, 12, and 24 weeks, as well as the dropout and satisfaction rates between patients in the two treatment arms.

## Methods

### Study Design

The STEM study design is a mixed methods, hybrid type II implementation-effectiveness trial. This design evaluates the effectiveness of an implementation strategy or intervention, while also evaluating the implementation context associated with the intervention [38,39]. This study is a randomized noninferiority trial testing the outcomes for email messaging (as a strategy for delivering daily evidence-based supportive messages to patients with MDD in addition to usual care), in comparison to outcomes for text messaging, which has been deemed effective by previous studies (with noted implementation limitations [28,33]). The relevant guidelines and checklist were obtained from the Enhancing the Quality and Transparency of Health Research (EQUATOR) network website and used in the design of this trial [40]. These include the SPIRIT (Standard Protocol Items: Recommendations for Interventional Trials) checklist (Multimedia Appendix 1) and CONSORT (Consolidated Standards of Reporting Trials) guidelines (Multimedia Appendix 2) [41,42].

### Study Settings

Patients will be recruited from the Alberta Health Services Access 24/7 clinic located in Edmonton, Canada. This city center Access 24/7 clinic is a zone-wide centralized intake clinic for addiction and mental health. Patients with mental health concerns can self-present or are referred by a primary care provider to the clinic for assessments and referral to other community mental health clinics for follow-up or to other community support services as necessary.

### Participant Recruitment

Patients who have been assessed by a psychiatrist at the Urgent Psychiatric Clinic of the Access 24/7 clinic in Edmonton and diagnosed with an MDD according to the Diagnostic and Statistical Manual of Mental Disorders (DSM-5) criteria using a structured clinical interview will be offered an information leaflet about the study and invited to participate.

### Inclusion Criteria

The inclusion criteria are as follows:

1. Persons aged 18 to 65 years who have the capacity to provide informed consent;
2. Patients who have been assessed using structured clinical interviews for DSM-5 and diagnosed with an MDD;
3. Patients who have a mobile phone with an active line and a functional email address and can access both email messages and text messages;
4. Patients who willingly agree to be enrolled in the trial and sign the consent form.

### Exclusion Criteria

Patients will be ineligible if they are patients with active psychotic disorders or residing outside of regular mobile phone and internet connection areas. They will also be ineligible if they previously or currently subscribed to Text4Hope, Text4Mood, Text4Support, or another supportive text messaging program.

### Sample Size

Based on the effect sizes (Cohen *d* of 0.85 and 0.67) achieved in 2 previous randomized trials (n<80 for each trial), which compared daily supportive text messages plus treatment as usual with treatment as usual alone for the management of patients with MDD, a sample size of 80 is estimated to have sufficient power for the current protocol. Allowing for a projected maximal 20% overall dropout rate, we propose to recruit 100 patients with MDD into the trial, with 50 patients randomly allocated into each study arm.

### Randomization and Blinding

We will use computer-generated block randomization to ensure balance (1:1) between the two treatment groups. Randomization codes will be provided via text message directly to the blinded researcher’s password-protected phone line with a secure online backup. This will occur for enrollment as each participant signs the consent form. All study participants will complete baseline assessments before randomization, and follow-up assessments will occur through online surveys with the survey links delivered via text messaging or email messaging depending on which study arm participants belong to. Participants will be asked to use either the email address or phone number with which they receive messages as their study ID on the follow-up online surveys.

### Intervention and Implementation Strategies

A research coordinator will assist participants who have provided written informed consent to enroll in either the email messaging or text messaging program by inputting their email address or phone number into the online email messaging or text messaging platforms that will be used to deliver the daily messages. Starting a day after enrollment, participants will receive either daily supportive text messages or daily supportive email messages. Both the email and text messages have the same content and have been crafted by mental health therapists, clinical psychologists, psychiatrists, and mental health service users based on cognitive behavior therapy principles. Each message will be scheduled to be delivered to the participants’ mobile phone or email address at 10 AM MT, and each participant will receive the messages for 6 months.

### Conceptual Framework

This hybrid trial will evaluate and compare both the effectiveness of each STEM strategy (supportive text messaging and email messaging) as well as context, process, and outcomes of the implementation. The trial will integrate two implementation science frameworks to guide the evaluation:

1. The RE-AIM (Reach, Effectiveness, Adoption, Implementation, and Maintenance) framework [43], which comprises five domains, will be used to assess external validity and feasibility of scale-up.
2. The Consolidated Framework for Implementation Research (CFIR) [43,44] will be used to systematically assess contextual factors (barriers and facilitators) that influence the adoption and implementation of the strategies. The CFIR guides the evaluation of contextual influences on intervention implementation and consists of five domains: intervention characteristics, outer setting, inner setting, characteristics of individuals, and process of implementation.

**Figure 1:**
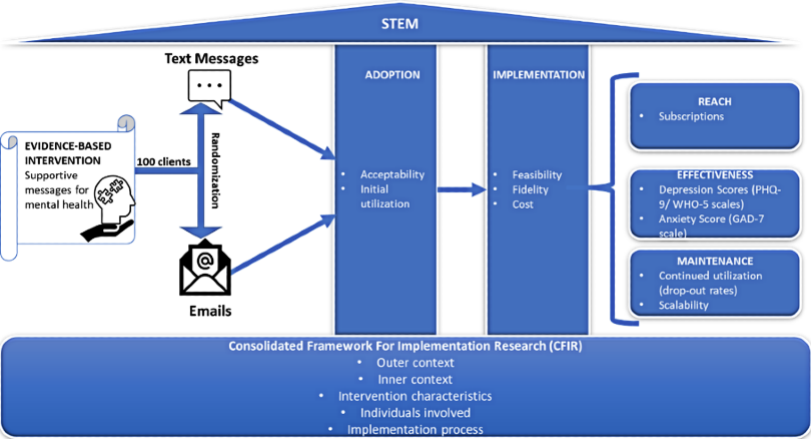
Conceptual framework of the *Supportive Text vs Email Messaging* (STEM) trial informed by the RE-AIM (Reach, Effectiveness, Adoption, Implementation, and Maintenance) and Consolidated Framework for Implementation Research frameworks. PHQ-9: Patient Health Questionnaire-9, WHO-5: 5-item World Health Organization Well-Being Index, GAD-7: Generalized Anxiety Disorder-7.

Figure 1 is an illustration of the evaluation framework of the STEM trial, while Table 1 is an overview of the outcome measures.

**Table 1:** Overview of the *Supportive Text vs Email Messaging* (STEM) outcome measures.

RE-AIM^a^ domain and outcomes			Measures		Data source		Timeline (months)										
						1-3		4-6		7-9		10-12		13-15		16-18	
**Reach**																	
	Dose delivered	The frequency of emails and text messages sent to the subscribers (including delivery failures)		STEM metadata		✓		✓		✓		✓		✓		✓	
	Dose received	The average number of times participants read the emails or text messages		Survey questionnaire						✓		✓		✓		✓	
**Effectiveness**																	
	Depression symptom score	Patient Health Questionnaire-9 (PHQ-9)		Clinical questionnaire						✓		✓		✓		✓	
	Patients’ quality of life	5-item World Health Organization Well-Being Index (WHO-5)		Clinical questionnaire						✓		✓		✓		✓	
	Anxiety symptom scores	Generalized Anxiety Disorder-7 (GAD-7)		Clinical questionnaire						✓		✓		✓		✓	
**Adoption**																	
	Acceptability/uptake	The number of subscriptions to the text and email services		STEM metadata												✓	
	Acceptability/uptake	Barriers and facilitators		Qualitative focus group discussions guided by the CFIR^b^												✓	
**Implementation**																	
	Feasibility	Implementation drivers (barriers and facilitators)		Qualitative focus group discussions guided by the CFIR												✓	
	Fidelity	The proportion of participants who read the messages at least once a day		Survey questionnaire						✓		✓		✓		✓	
	Cost	Comparative cost of developing and maintaining both strategies		STEM metadata		✓		✓		✓		✓		✓		✓	
**Maintenance**																	
	Sustainability	Dropout rates; barriers and facilitators		Subscription data; qualitative focus group discussions guided by the CFIR												✓	

^a^RE-AIM: Reach, Effectiveness, Adoption, Implementation, and Maintenance.

^b^CFIR: Consolidated Framework for Implementation Research.

### Effectiveness Evaluation

#### Hypothesis

As the same messages will be delivered through the supportive text message program and the email program, we hypothesize that daily email messaging will not be inferior to daily supportive text messaging in reducing depression symptoms and improving quality of life for patients with MDD. We further hypothesize that participant dropout rates and satisfaction rates will be comparable in the two arms of the study.

#### Data Collection

Participants who provide informed written consent and agree to participate in the study will be asked to complete a baseline assessment questionnaire that captures both demographic and clinical information. At 6, 12, and 24 weeks, an online survey link will be sent to participants via email or text messages along with an invitation for them to complete the assessments. Participants will enter the email address or phone number through which they receive the messages as their study identifier at the start of the survey so that their data at the 4 time points can be matched. Participants who have not completed their follow-up assessments within 3 days of the due date will be contacted by a research coordinator and reminded to do so, or if they require assistance to complete the assessment, such assistance will be offered, including the option to complete the assessment over the telephone. In instances where such assistance is required, a blinded research assistant will be asked to read out the survey questions to the participant and record their responses.

#### Outcome Measures

The primary effectiveness outcome measures for the study will be the differences between mean scores on the following scales measured at baseline, 6, 12, and 24 weeks for the two study arms: the PHQ-9, WHO-5, and GAD-7.

The PHQ-9 scale will be used to provide depression symptom scores [35]. The PHQ-9 is a 9-item validated instrument (associated with a Cronbach alpha of .89) that is used to diagnose and measure the severity of depression in general medical and mental health settings. Each of the 9 questionnaire items is scored between 0 (not at all) to 3 (nearly every day). Higher scores on the scale indicate higher levels of depression. The PHQ-9 demonstrated good convergent validity with related constructs with an adequate internal consistency [45].

The WHO-5 will be used to measure patients’ quality of life [46]. The WHO-5 is a short 5-item questionnaire that measures the subjective well-being of the respondents. The scale has adequate validity as an outcome measure in clinical trials and as a generic scale for assessing well-being over time or between groups [47].

Secondary outcomes include changed mean scores on the GAD-7 scale [37] at baseline, 6, 12, and 24 weeks for the two study arms, as well as participant dropout rates and satisfaction rates at 12 and 24 weeks in the two arms. The GAD-7 is a validated 7-item questionnaire (associated with a Cronbach alpha of .92) that is used to assess the self-reported levels of anxiety in respondents in the 2 weeks prior to assessment. Each item on the scale is scored between 0 (not at all) to 4 (nearly every day). Higher scores on the scale indicate higher levels of anxiety.

#### Data Analyses

We will summarize the baseline demographic and clinical characteristics of participants in raw values and percentages and compare them between the two treatment arms using chi-square or Fisher exact tests and appropriate *t* tests. The primary and secondary outcome measures will be analyzed using descriptive and inferential statistical analysis. For the primary outcome measures, we will use analysis of covariance (ANCOVA) to assess the change in scores between the two treatment arms, with baseline PHQ-9 and WHO-5 scores as covariates; the treatment arm as the independent variable; and PHQ-9 and WHO-5 scores at 6, 12, and 24 months as respective dependent variables for each time point. Secondary quantitative outcome measures will be compared using chi-square or Fisher exact tests and appropriate *t* tests.

### Implementation Evaluation

Implementation evaluation will seek to explore and compare the context, process, and outcomes of implementing the two strategies, guided by the CFIR and the RE-AIM frameworks. A mixed method approach will be applied in evaluating the implementation of the STEM trial.

#### Data Collection

Quantitative data will be collected on the implementation outcomes: acceptability, fidelity (adherence to reading messages), and sustainability (continued utilization). Data will be collected using user satisfaction survey questionnaires at 6, 12, and 24 weeks for the two study arms.

Qualitative data will be collected via focus group (see details below) discussions involving the patients. The discussion topic guide will be informed by the CFIR domains and will also elicit participants’ views on barriers and facilitators to the implementation of both strategies.

We will invite a random sample of 10 participants via text message and email to take part in a virtual focus group workshop using end-to-end encrypted teleconferencing (with appropriate consent for confidentiality) to discuss the impacts of the interventions. In order to explore the reasons why participants remain or drop out of the study, we will hold a focus group for a cross-section of participants who remained in the study and those who dropped out to discuss the impacts of the supportive email and text message interventions. Thus, an invitation will go to both those who remain in the study and those who dropped out, and participation in the focus group workshop will be voluntary. We will offer a CAD $50 (US $39) gift card as honorarium to cover the cost for participants attending the focus group.

#### Data Analysis

Quantitative data will be analyzed as described under “Effectiveness Evaluation” section. Qualitative data obtained through audio recordings from the patient focus group discussions will be transcribed, then analyzed using thematic analysis aided by NVIVO software (QSR International). Data analysis will be both deductive and inductive. Deductive analysis will be guided by the CFIR and RE-AIM. Two researchers will independently conduct the initial coding after familiarization with the transcripts; thereafter, the subsequent and final coding criteria will be agreed upon by both researchers following an iterative review of emerging themes as informed by the research questions. The final sets of themes and subthemes relevant to the overall research goals will be reported and supported with verbatim quotes.

##### Addressing Missing Data

Though preventing missing data is difficult in clinical research, measures will be put in place by the researchers to minimize the occurrence of missing data. This includes having a well-defined data collection strategy that is understood by all study personnel, as well as having a simple set of questionnaires to collect only relevant data needed, minimizing the number of follow-ups with participants, and creating substantial gaps in the number of follow-ups. The investigators will identify and actively engage the participants who would be at the greatest risk of being lost during follow-up. Further, the data collection would be monitored and reported in as close to real time as possible during the study. Notwithstanding the above, the mean substitution strategy would be employed by the research team to handle missing data [48]. Here, the mean value of a variable is used in the place of the missing data value for that same variable. This allows the researchers to utilize the collected data in an incomplete data set. Questionnaires with more than 20% missing responses will be excluded.

##### Gender Analysis

Gender analyses will guide the entire study. Beyond gender disaggregation and adjustments to accommodate potential differences in subscription, the development and adaptation of the supportive messages will receive input from gender specialists. Further, the implementation science framework CFIR is designed to accommodate and understand gender among the characteristics of individuals involved in intervention implementation.

### Ethics and Dissemination

All study participants will be provided with an information leaflet and offered the opportunity to ask questions about the study before being asked to provide written informed consent to participate in the study. Data collection will occur online through patient self-completed rating scales. The study has received institutional review board approval from the University of Alberta Human Ethics Review Board (Pro00105429), and the trial has been registered at ClinicalTrials.gov (NCT04638231). Results will be disseminated as publications, conference presentations, and stakeholder policy dialogues.

## Results

Data collection for this trial began in April 2021. We expect the study results to be available within 18 months of study commencement. The findings of the trial are expected to shed light on the feasibility, acceptability, and effectiveness of using automated emails as a strategy for delivering supportive messages to patients with MDD in comparison to text messaging.

## Discussion

### Implications of the Study

Supportive messaging may be effective for providing patient support for various mood disorders. It is accepted that this intervention can reduce demand for physical mental health services, particularly the need to seek acute mental health care at emergency departments [28,30,33].

As previous studies have delivered these messages using text messaging services on mobile devices, this study seeks to examine the effectiveness of email-based delivery of equivalent messages. The STEM trial will provide comparative evidence on both delivery strategies as well as useful information to inform us on how to better implement them at scale. Findings from this study will be useful for mental health professionals managing MDD, and will contribute to knowledge on eHealth approaches generally. During the current COVID-19 pandemic, this is particularly important as health care agencies seek alternatives to physical contact where possible [49,50].

The external validity of this trial is limited by the single catchment site context. Despite this, findings from this trial will inform the implementation of a planned full-scale multicenter clinical trial using Resilience N Hope [51] to determine the effectiveness of this strategy across various contexts. The limitations are mitigated by the mixed methods approach to the design. It is hoped that the triangulation of findings from both quantitative and qualitative methods will provide useful insights. In addition, the proposed application of implementation science frameworks will provide practical information to inform implementation at scale. Unavoidably, the study sample might experience a considerable selection bias, based upon the necessity of having a functioning mobile phone and an email address in order to be able to participate in the study. Thus, this bias may limit the generalizability of our findings as patients who lack these prerequisites will not necessarily be represented in here.

### Conclusion

The STEM trial is expected to inform effective and contextually feasible strategies for delivering supportive messages to patients with MDD. The outcome of this trial will have translational impact on routine patient care and access to mental health as well as potentially support mental health policy decision-making for health care resource allocation.

## Appendix

Multimedia Appendix 1 The SPIRIT checklist.

Multimedia Appendix 2 CONSORT 2010 checklist.

## Abbreviations

ANCOVA: analysis of covariance
BDI-II: Becks Depression Inventory-II
CFIR: Consolidated Framework for Implementation Research
CONSORT: Consolidated Standards of Reporting Trials
DSM-5: Diagnostic and Statistical Manual of Mental Disorders
EQUATOR: Enhancing the Quality and Transparency of Health Research
GAD-7: Generalized Anxiety Disorder-7
MDD: major depressive disorder
PHQ-9: Patient Health Questionnaire-9
RE-AIM: Reach, Effectiveness, Adoption, Implementation, and Maintenance
SPIRIT: Standard Protocol Items: Recommendations for Interventional Trials
STEM: Supportive Text vs Email Messaging
WHO-5: 5-item World Health Organization Well-Being Index
