# Correction: Text Messaging Versus Email Messaging to Support Patients With Major Depressive Disorder: Protocol for a Randomized Hybrid Type II Effectiveness-Implementation Trial

**DOI:** 10.2196/34515

**Published:** 2021-11-08

**Authors:** Medard Kofi Adu, Reham Shalaby, Ejemai Eboreime, Adegboyega Sapara, Nnamdi Nkire, Rajan Chawla, Chidi Chima, Michael Achor, Felix Osiogo, Pierre Chue, Andrew J Greenshaw, Vincent Israel Agyapong

**Affiliations:** 1 Department of Psychiatry Faculty of Medicine and Dentistry University of Alberta Edmonton, AB Canada; 2 Department of Psychiatry Faculty of Medicine Dalhousie University Halifax, NS Canada

In “Text Messaging Versus Email Messaging to Support Patients With Major Depressive Disorder: Protocol for a Randomized Hybrid Type II Effectiveness-Implementation Trial” (JMIR Res Protoc 2021;10(10):e29495) the authors noted two errors.

1. In the originally published paper, author affiliations 1 and 2 were numbered incorrectly. The numbering of these affiliations has been switched in the corrected paper.

2. The equal contribution symbol '*' for author Felix Osiogo has been removed.

The complete list of authorship and affiliations was originally published as follows:

Medard Kofi Adu^1*^, BSc, MSc; Reham Shalaby^1*^, MD; Ejemai Eboreime^1^, MD, PhD; Adegboyega Sapara^1^, PhD, FRCPC; Nnamdi Nkire^1^, MD, MBBS, DHSM, DCP, FRAMI; Rajan Chawla^1^, MBBS, CCT, MSC, MRCPsych; Chidi Chima^1^, MBBS, MPH, PhD; Michael Achor^1^, MD, MSC, FRCPC; Felix Osiogo^1*^, FRCPC, FWACS, CCT-UK, MBBS; Pierre Chue^1^, MD, DABPN, MRCPsych, FRCPC; Andrew J Greenshaw^1^, PhD, FRSA; Vincent Israel Agyapong^1,2^, MD, PhD, FRCPC, FRCPsych

^1^Department of Psychiatry, Faculty of Medicine, Dalhousie University, Halifax, NS, Canada

^2^Department of Psychiatry, Faculty of Medicine and Dentistry, University of Alberta, Edmonton, AB, Canada

^*^these authors contributed equally

This has been corrected to the following:

Medard Kofi Adu^1*^, BSc, MSc; Reham Shalaby^1*^, MD; Ejemai Eboreime^1^, MD, PhD; Adegboyega Sapara^1^, PhD, FRCPC; Nnamdi Nkire^1^, MD, MBBS, DHSM, DCP, FRAMI; Rajan Chawla^1^, MBBS, CCT, MSC, MRCPsych; Chidi Chima^1^, MBBS, MPH, PhD; Michael Achor^1^, MD, MSC, FRCPC; Felix Osiogo^1^, FRCPC, FWACS, CCT-UK, MBBS; Pierre Chue^1^, MD, DABPN, MRCPsych, FRCPC; Andrew J Greenshaw^1^, PhD, FRSA; Vincent Israel Agyapong^1,2^, MD, PhD, FRCPC, FRCPsych

^1^Department of Psychiatry, Faculty of Medicine and Dentistry, University of Alberta, Edmonton, AB, Canada

^2^Department of Psychiatry, Faculty of Medicine, Dalhousie University, Halifax, NS, Canada

^*^these authors contributed equally

The correction will appear in the online version of the paper on the JMIR Publications website on November 5, 2021, together with the publication of this correction notice. Because this was made after submission to PubMed, PubMed Central, and other full-text repositories, the corrected article has also been resubmitted to those repositories.

