# Supplementary material for: Text Messaging Versus Email Messaging to Support Patients With Major Depressive Disorder: Protocol for a Randomized Hybrid Type II Effectiveness-Implementation Trial
Source: JMIR Res Protoc. 2021 Oct 13;10(10):e29495. doi: 10.2196/29495 (PMC8552095; doi:10.2196/29495)
Supplement: Multimedia Appendix 1 [file resprot_v10i10e29495_app1.docx]

**Multimedia Appendix 1. The SPIRIT checklist.**

Standard Protocol Items: Recommendations for Interventional Trials (SPIRIT) flow diagram for each patient.

|  | **STUDY PERIOD**  **(Months)** | | | | | | | | | | | | | | |
| --- | --- | --- | --- | --- | --- | --- | --- | --- | --- | --- | --- | --- | --- | --- | --- |
|  | **Enrolment** | **Allocation** | **Post-allocation** | | | | | | | | | | | | **Close-out** |
| **TIMEPOINT** | **0** | **0** | **1** | **2** | **3** | **4** | **5** | **6** | **7** | **8** | **9** | **10** | **11** | **12** | **12-18** |
| **ENROLMENT:** |  |  |  |  |  |  |  |  |  |  |  |  |  |  |  |
| **Eligibility screen** | X |  |  |  |  |  |  |  |  |  |  |  |  |  |  |
| **Informed consent** | X |  |  |  |  |  |  |  |  |  |  |  |  |  |  |
| **Allocation & subscription** |  | X |  |  |  |  |  |  |  |  |  |  |  |  |  |
| **INTERVENTIONS:** |  |  |  |  |  |  |  |  |  |  |  |  |  |  |  |
| ***Daily supportive text messages*** |  |  | X | X | X | X | X | X |  |  |  |  |  |  |  |
| ***Daily supportive email messages*** |  |  | X | X | X | X | X | X |  |  |  |  |  |  |  |
| **ASSESSMENTS:** |  |  |  |  |  |  |  |  |  |  |  |  |  |  |  |
| ***WHO-5 Index*** | X |  |  | X | X |  |  | X |  |  |  |  |  |  |  |
| ***PHQ-9 score*** | X |  |  | X | X |  |  | X |  |  |  |  |  |  |  |
| ***GAD-7 Index*** | X |  |  | X | X |  |  | X |  |  |  |  |  |  |  |
| ***Implementation evaluation*** | X | X | X | X | X | X | X | X |  |  |  |  |  |  |  |
| ***FGD on experiences*** |  |  |  |  |  |  |  |  |  |  |  |  |  |  | X |
| ***Satisfaction survey*** |  |  |  |  |  |  |  |  | X |  |  |  |  |  |  |
